# iFORM: Incorporating Find Occurrence of Regulatory Motifs

**DOI:** 10.1371/journal.pone.0168607

**Published:** 2016-12-19

**Authors:** Chao Ren, Hebing Chen, Bite Yang, Feng Liu, Zhangyi Ouyang, Xiaochen Bo, Wenjie Shu

**Affiliations:** Department of Biotechnology, Beijing Institute of Radiation Medicine, Beijing, China; INSERM U869, FRANCE

## Abstract

Accurately identifying the binding sites of transcription factors (TFs) is crucial to understanding the mechanisms of transcriptional regulation and human disease. We present incorporating Find Occurrence of Regulatory Motifs (iFORM), an easy-to-use and efficient tool for scanning DNA sequences with TF motifs described as position weight matrices (PWMs). Both performance assessment with a receiver operating characteristic (ROC) curve and a correlation-based approach demonstrated that iFORM achieves higher accuracy and sensitivity by integrating five classical motif discovery programs using Fisher’s combined probability test. We have used iFORM to provide accurate results on a variety of data in the ENCODE Project and the NIH Roadmap Epigenomics Project, and the tool has demonstrated its utility in further elucidating individual roles of functional elements. Both the source and binary codes for iFORM can be freely accessed at https://github.com/wenjiegroup/iFORM. The identified TF binding sites across human cell and tissue types using iFORM have been deposited in the Gene Expression Omnibus under the accession ID GSE53962.

## Introduction

Gene regulation is co-ordinately regulated by interactions of many transcription factors (TFs), many of which bind promoter and enhancer DNA preferentially at characteristic sequence ‘motifs’. Motifs are short patterns described as position weight matrices (PWMs) that tend to be conserved by purifying selection. Identifying and understanding these TF motifs can provide critical insight into the mechanisms of transcriptional regulation and human disease. However, accurate identification of these binding site motifs is still challenging because a single TF will often recognize a variety of similar sequences.

Over the past several decades, many classical algorithms have been developed to discover DNA regulatory motifs. FIMO [1], Consensus [2, 3], and STORM [4] function solely as motif scanners, whereas RSAT [5] and HOMER [6] provide multiple functions for analysing regulatory sequences in general. S1 Table summarizes the features of iFORM and these five algorithms as motif scanners. Each of these methods has its own merits for identifying potential TF binding; however, it is still a major challenge to integrate superiorities and to preclude inferiorities of these complementary methods. Several studies have demonstrated that higher accuracy and sensitivity can be achieved by incorporating multiple motif discovery programs [7–9]; however, this approach will result in considerable computational overhead for scoring the large number of discovered motif instances obtained with different programs.

We describe incorporating Find Occurrence of Regulatory Motifs (iFORM), a software tool for scanning DNA sequences with TF motifs by integrating five classical motif discovery methods. We used Fisher’s combined probability test to convert the resulting *p*-values obtained from the five methods to a χ^2^ statistic, which follows a chi-squared distribution. We then applied false discovery rate analysis to estimate a *q*-value for each motif instance. By systematically assessing the accuracy of prediction performance, iFORM achieves higher accuracy and sensitivity relative to the five classical algorithms. Although iFORM integrates five methods, it is efficient, allowing for scanning sequences at a rate of 3.5 Mb/s on a single CPU. Additionally, we have illustrated the use of iFORM by producing high-quality genome-wide maps of transcription factor binding sites (TFBSs) for 542 TFs within DNaseI hypersensitive sites (DHSs) of 133 human cell and tissue types that were generated by the ENCODE Project [10] and the NIH Roadmap Epigenomics Mapping Consortium [11] in our recent studies. We also demonstrated the utility of iFORM to further reveal individual roles of functional elements in gene regulation and diseases based on these maps.

## Materials and Methods

### Data sets

DNaseI Hypersensitivity by Digital DNaseI data were obtained from both the ENCODE Project [10] and the NIH Roadmap Epigenomics Mapping Consortium [11]. TFs identified by ChIP-seq data were obtained from the ENCODE Project [10]. The uniform processing pipeline of the ENCODE Integrative Analysis Consortium was used to generate uniform peaks from both the DNase-seq and ChIP-seq data. The use of these data adheres strictly to the ENCODE and Roadmap Epigenomics Consortium Data Release Policy. PhastCons were extracted from the hg19 conservation track of the UCSC Genome Browser [12].

### iFORM

iFORM integrates the TF binding sites identified by five classic methods: FIMO [1], Consensus [2, 3], STORM [4], RSAT [5] and HOMER [6]. For each discovered TFBS, a combined *p*-value obtained from these five methods was calculated using Fisher's combined probability test. First, a test statistic was calculated using the following formula:

$$\chi^{2}=-2\sum_{i=1}^{5}{log}_{e}(p_{i})\sim \chi^{2}(10)$$

where *p*_i_ is the *p*-value calculated from the five methods for each TFBS and χ^2^ follows a chi-squared distribution χ^2^(10) when *p*_i_ is independent. Thus, a combined *p*-value can be assigned to this test statistic. Then, we used a bootstrap method [13] to estimate false-discovery rates (FDRs) and report for each *p*-value a corresponding *q*-value. Before applying Fisher's combined probability test to our iFORM, we collected different sets of *p*_i_ values derived from the five methods by scanning diverse TF motifs in diverse cells/tissues in the human genome to test the independence of *p*_i_ values derived from the five methods. A Hilbert-Schmidt independence criterion (HSIC) method was used to test the independence jointly [14]. The threshold and HSIC score were calculated using *d*-variable HSIC (dHSIC) R-packages [15], with alpha level 0.05. The independent hypothesis was rejected if and only if threshold was equal or less than HSIC score. Our result demonstrated that the thresholds were strictly greater than HSIC scores in all the collected *p*_i_ value sets, suggesting the joint independence of the *p*_i_ values derived from the five methods (S2 Table). Thus, Fisher's combined probability test can be applied to our iFORM. iFORM can be freely accessed at https://github.com/wenjiegroup/iFORM.

### Generation of “gold-standard” data

To validate the predicted TFBSs using the ROC (receiver operating characteristic) approach, we applied a method similar to that presented in a previous study [16] to generate “gold-standard” data for a few TFs. Briefly, we scanned the genomic sequences under the uniform DHSs in the hg19 genome using the five methods incorporated by iFORM with default parameters for each TF motif in the “gold-standard” set separately. Then, for each TF, all motif instances (*p*-value < 10^−9^) located within the corresponding TF ChIP-seq peaks were considered the set of TFBS positives. The set of TFBS negatives was defined as all motif instances (*p*-value < 10^−9^) that did not overlap with a ChIP-seq peak and had a greater fraction of total mapped reads from the “Control” compared to the ChIP-seq treatment. In total, there were 103 sets of “gold-standard” data for 12 TFs from 51 cells/tissues (S3 Table).

### Assessing the performance of iFORM

The prediction performance of iFORM was assessed using three methods, including ROC curve analysis, precision-recall curve analysis and a correlation-based approach. First, we used ROC curves and the area under the curve (AUC) to assess the accuracy of the prediction performance of iFORM. The ROC approach assumes that there are “gold-standard” data, including TFBS positives and TFBS negatives, that can be used for comparison to confidently evaluate our predictions of TF binding for a subset of the candidate motif sites. Furthermore, we also adopted precision-recall curves to assess the imbalance of the “gold-standard” data sets.

In addition to the ROC curve analysis, which classifies the motif instances as either TFBS positive or TFBS negative, we adopted a correlation approach that takes into account all locations except those in or near repetitive regions. The correlation approach assumes that the larger the correlation between the predicted value and the ChIP-seq signal and the smaller the correlation with the “control” background noise, the better the prediction accuracy. We extracted the ChIP-seq reads and the “control” reads around each motif site, and we used the Pearson correlation to measure the association between the total number of reads and the scores reported by iFORM and the five classical methods.

## Results and Discussion

### Implementation of iFORM

**Fig 1:**
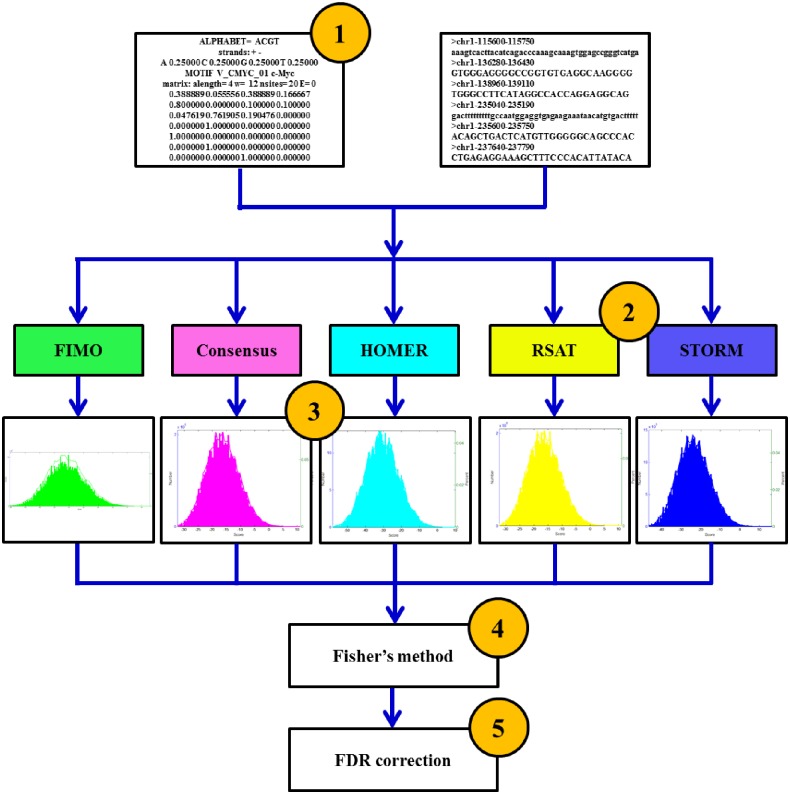
The workflow of iFORM. Overview of the iFORM workflow. (1) Assemble input data. The results may be improved by restricting the input to high-confidence sequences. Some programs achieve improved performance using phylogenetic conservation information from orthologous sequences or information about protein DNA-binding domains. (2) Choose several motif discovery programs for the analysis. (3) Test the statistical significance of the resulting motifs. Use control calculations to estimate the empirical distribution of scores produced by each program on random data. (4) For each identified TFBS, a combined *p*-value obtained from these five methods was calculated using Fisher's combined probability test. (5) FDR correction of the combined *p*-values obtained from Fisher’s method.

iFORM integrated the motif instances identified by the five classical algorithms, FIMO [1], Consensus [2, 3], STORM [4], RSAT [5] and HOMER [6], based on Fisher’s method. We chose these five methods due to their great popularity in applications and the great generality of their scoring methods as motif scanners. iFORM was built using C, and an overview of the workflow is illustrated in Fig 1. To improve efficiency, we extracted the core source code of the motif discovery functions of these five algorithms and integrated them into the framework of iFORM instead of simply combining the resulting *p*-values obtained from these algorithms.

iFORM accepts DNA sequences in FASTA format or genomic coordinates in BED or GFF formats, and it takes as an input one or more TF motifs (represented as PWMs) that can be collected from an existing motif database, generated the MEME algorithm, or even be defined by the user. For each motif instance, iFORM computes a χ^2^ statistic that was obtained by combining *p*-values resulting from the five algorithms using Fisher's method, and it converts these statistics to *p*-values using the chi-squared distribution. Finally, iFORM uses a bootstrap method [13] to estimate FDRs and reports for each *p*-value a corresponding *q*-value, which is defined as the minimal FDR threshold at which the *p*-value is deemed significant [17].

iFORM outputs a ranked list of motif instances, each with an associated χ^2^ score, *p*-value and *q*-value. The list is illustrated as an HTML report, as an XML file in CisML format [18], as a plain text file and as tab-delimited files in gff formats suitable for input in the UCSC Genome Browser [12].

### Performance assessment of iFORM using “gold-standard” data

**Fig 2:**
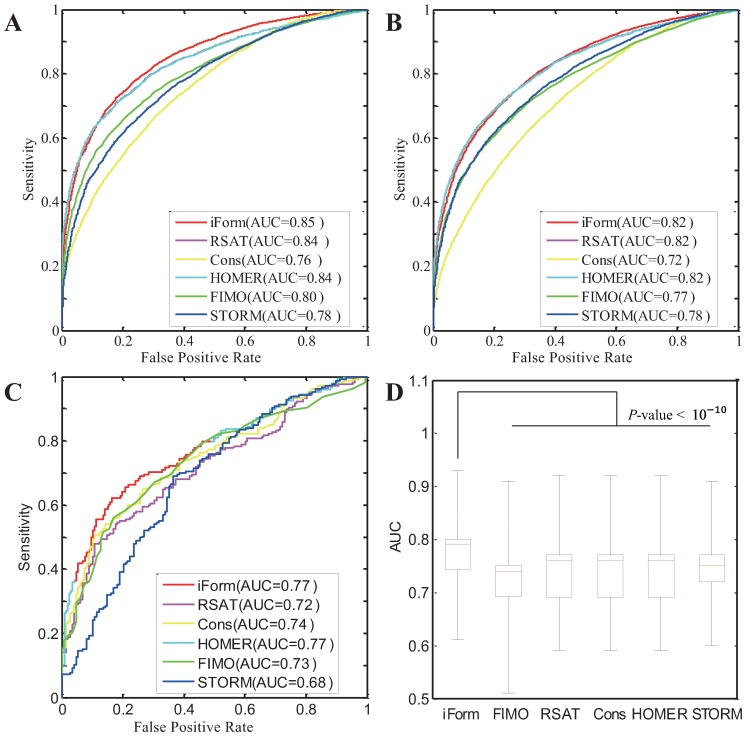
Performance assessment of iFORM using ROC curves. (A–C) ROC curves of multiple algorithms using “gold-standard” data from (A) CTCF in GM12878 cells provided in a previous study; (B) CTCF in GM12878 cells that was generated in our study. (C) REST in H1 cells. (D) Boxplot distributions of AUC for diverse TFs in multiple cells/tissues.

Because iFORM integrates five classical algorithms based on Fisher’s method, it is expected that iFORM can achieve higher accuracy and sensitivity compared with the five methods. To test this expectation, we first examined the CTCF motif instances identified by these six methods separately. We found that many of the CTCF binding sites, which are well annotated by DNase-seq, DNaseI digital genomic footprint (DGF), and corresponding TF ChIP-Seq data in H1 cells, can only be discovered by iFORM, not the other five classical methods (Figure A in S1 File). To systematically assess the accuracy of prediction performance for each motif instance, we used ROC curves and the corresponding AUC and precision-recall curves on the “gold-standard” data of six TF ChIP-seq data in GM12878 cells provided in a previous study [16] (Fig 2A and Figure C and D in S1 File). Our results suggest that our iFORM method showed higher AUC values than those of the other five classical methods.

To further validate the predicted TF binding sites, we used the method presented by Roger Pique-Regi et al. [16] to generate “gold-standard” data for many TF ChIP-seq sequences that were produced from different laboratories and from different cells/tissues (S3 Table). Using our newly generated “gold-standard” data of CTCF in GM12878 cells, we obtained ROC curves and precision-recall curves similar to those presented previously (Fig 2B and Figure C in S1 File). In addition, similar ROC curves and precision-recall curves were also obtained for the newly generated “gold-standard” data of CTCF in GM12878 produced from different labs (Fig 2C) and for the newly generated “gold-standard” data of CTCF in other cells (Figure C and D in S1 File). Furthermore, we assessed the prediction performance using new “gold-standard” data from 103 sets of TF ChIP-seq data produced in the ENCODE project (S3 Table). Across these “gold-standard” datasets, our iFORM achieves significantly higher AUCs than the other five algorithms (Fig 2D and S3 Table, *p*-value < 10^−10^, Kolmogorov–Smirnov test). These results suggest that iFORM manifested superior performance consistency in different laboratories and different cells/tissues across multiple TFs, and it demonstrated higher accuracy and sensitivity compared with the five classical methods.

**Fig 3:**
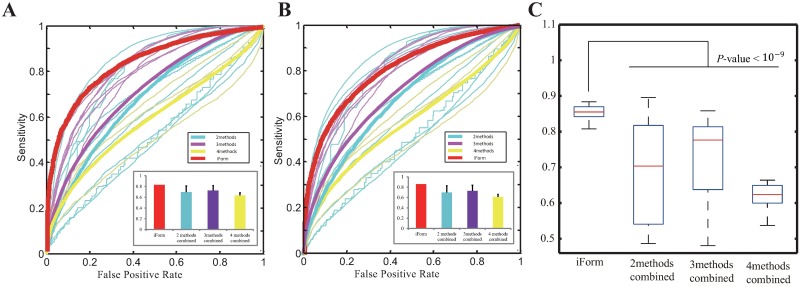
Performance comparisons between iFORM and integrating methods. (A-B) Performance comparisons between iFORM and integrating methods. ROC curves of iFORM and that of integrating only two, three, or four methods based on “gold-standard” data CTCF in GM12878 cells provided in a previous study (A) and the “gold-standard” data CTCF in GM12878 cells generated in this study (B). (C) Boxplot distributions of AUC of iFORM and of integrating only two, three, or four methods for diverse TFs in multiple cells/tissues.

Furthermore, we examined whether the performance of iFORM was superior to that of integrating only two, three, or four methods based on “gold-standard” data in GM12878 cells provided in a previous study [13] (Fig 3A) and the “gold-standard” data generated in this study (Fig 3B). Again, our iFORM achieved significantly higher AUCs compared with integrating only two, three, or four methods across these “gold-standard” datasets (Fig 3C and S3 Table, *p*-value < 10^−9^, two-sample Kolmogorov–Smirnov test). Taken together, our results convincingly demonstrate that iFORM presents superior performance compared with the five classical methods as well as integrating only two, three, or four methods.

### Performance assessment of iFORM using a correlation approach

**Fig 4:**
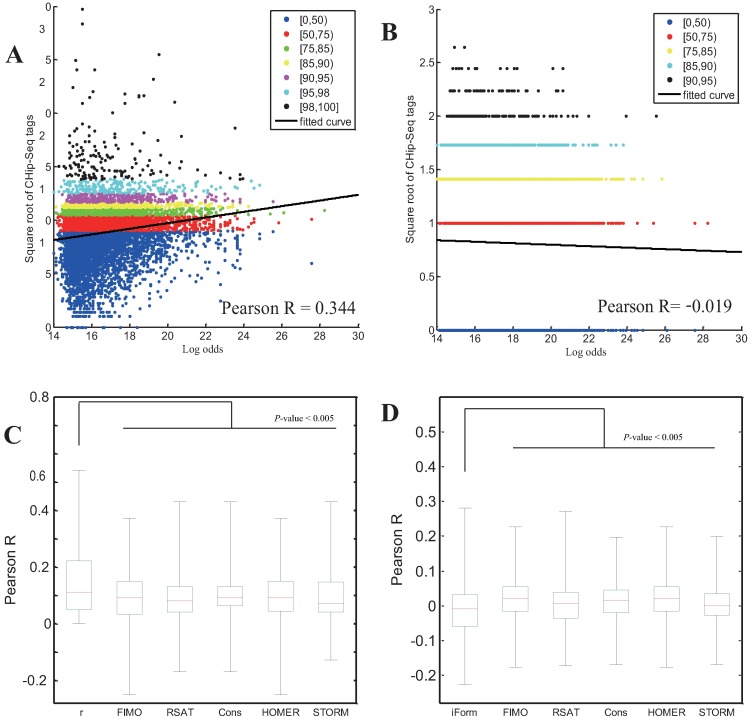
Performance assessment of iFORM using correlation methodology. (A-B) Scatter plot of the correlation between the log of odds from iFORM and the square-root transformed count of ChIP-seq (A) and “control” (B) reads in the 400 bp region surrounding each CTCF motif instance. (C–D) Performance comparison between iFORM and currently available methods. Boxplot distribution of Pearson correlations between the log of odds from iFORM and the square-root transformed count of ChIP-seq (A) and “control” (B) reads for diverse TFs in multiple cells/tissues.

Thus far, we have validated iFORM with ROC analysis, which requires a binary classification of TFBSs as positive/negative using ChIP-seq as a “gold standard”. However, it is becoming increasingly evident that TF binding is not perfectly dichotomous (bound or unbound); in many cases, quantitative partial binding occurs, which corresponds to the fraction of cells that have TF binding at a particular site at any given time [19, 20]. Indeed, our iFORM method is formulated as a combined χ^2^ statistic, which could quantitatively reflect the level of TF occupancy to some extent. Thus, we presumed that correlations of the statistical scores with the ChIP-seq signal and with the “control” background noise could be used to validate iFORM. The larger the former value and the smaller the latter value, the better the prediction accuracy of iFORM. Applying this procedure to the other five classical methods, we found that iFORM presented substantially superior performance relative to the other methods (Fig 4A and 4B). Across the multiple TFs that we tested, iFORM showed consistently superior accuracy compared with the other methods (Fig 4C and 4D and S5 Fig, *p*-value < 0.005, two-sample Kolmogorov–Smirnov test). Taken together, these findings suggest that iFORM provides state-of-the-art performance relative to the five existing methods.

### Application of iFORM for identifying TFBSs across multiple cells/tissues

We collected PWMs of the 542 TFs corresponding to 796 motif models from the TRANSFAC [21], JASPAR [22], and UniPROBE [23] databases as described in our previous study [24–26]. We applied iFORM to produce high-quality genome-wide maps of the TFBSs for the 542 TFs within DHSs of 133 human cell and tissue types that were generated by the ENCODE Project [10] and the NIH Roadmap Epigenomics Mapping Consortium [11] (*p*-value < 10^−18^). On average, scanning DHSs of each cell/tissue took 30 min and 10 s of wall clock time on an Intel Xeon 2.2 GHz CPU, which is equivalent to scanning at a speed of 3.5 Mp/s.

Based on these data, we found that TFBSs were clustered together in the human genome, and we report the first comprehensive map of TFBS cluster regions across human cell and tissue types. An integrative analysis of these regions revealed a novel transcriptional regulation model of the accessible chromatin landscape [24]. Additionally, we investigated the HOT (high-occupancy target) regions, which were defined as TFBS cluster regions with extremely high TFBS complexity. We found that HOT regions play key roles in human cell development and differentiation [25]. Furthermore, we explored the prevalence of single nucleotide polymorphisms (SNPs) identified by genome-wide association studies (GWAS) in HOT regions, which demonstrated key roles of HOT regions in human disease and cancer [26]. These findings represent a critical step towards further understanding disease biology, diagnosis, and therapy.

## Conclusions

The iFORM tool presented in this study was designed to incorporate five classical regulatory motif discovery methods using Fisher’s method. iFORM is an easy-to-use and efficient motif discovery tool that achieves higher accuracy and sensitivity by integrating the results from multiple motif discovery programs. iFORM has provided accurate results using a variety of data from the ENCODE Project and the NIH Roadmap Epigenomics Project in our recent studies, and it has demonstrated its utility to further elucidate individual roles of functional elements in the mechanisms of transcriptional regulation and human disease.

## Supporting Information

S1 File This file contains all Supplementary figures (A-E). (PDF)

S1 Table Summaries of the five motif scanners. (DOCX)

S2 Table Independence test of *p*_i_ derived from the five methods. (XLSX)

S3 Table Performance assessment of iFORM. (XLSX)
