# Supplementary material for: iFORM: Incorporating Find Occurrence of Regulatory Motifs
Source: PLoS One. 2016 Dec 19;11(12):e0168607. doi: 10.1371/journal.pone.0168607 (PMC5167396; doi:10.1371/journal.pone.0168607)
Supplement: S1 File — (PDF) [file pone.0168607.s001.pdf]

Supplementary Figures

Figure A in S1 File. An example of a CTCF motif binding site

An example of a CTCF motif binding site, which is well annotated by DNase-seq, DNaseI digital genomic footprint (DGF), and corresponding TF ChIP-Seq data in H1 cells, that can only be discovered by iFORM and not by the other five classical methods.

Figure B in S1 File. ROC curves of five TFs from a previous study

ROC curves of multiple algorithms using “gold-standard” data for five TFs that were generated in a previous study. (A) REST. (B) GABPA. (C) SRF. (D) MAX. (E) JUND.

Figure C in S1 File. Precision-recall curves of diverse TFs across different cells/tissues from different labs

Precision-recall curves of multiple algorithms using “gold-standard” data for three TFs from diverse human cells/tissues. (A) CTCF in GM12878 cells provided in a previous study; (B) CTCF in GM12878 cells that was generated in our study. (C) GABPA provided in a previous study. (D) SRF provided in a previous study. (E) CTCF in GM12878 cells as generated from a Broad Institute lab. (F) CTCF in H1 cells as generated from a university of Washington (UW) lab.

Figure D in S1 File. ROC curves of three TFs across diverse cells/tissues from different labs

ROC curves of multiple algorithms using “gold-standard” data for three TFs from diverse human cells/tissues. (A) REST in K562 cells as generated from a Broad Institute lab. (B) CTCF in GM12878 cells as generated from a Broad Institute lab. (C) CTCF in H1 cells as generated from a university of Washington (UW) lab.

Figure E in S1 File. Performance assessment of iFORM using correlation methodology

(A–E) Scatter plot of the correlation between the log of odds from FIMO (A), CONSENSUS (B), RSAT (C), HOMER (D), and STORM (E) with the square-root transformed count of ChIP-seq reads in the 400 bp region surrounding each CTCF motif instance. (F–J) Scatter plot of the correlation between the log of odds from FIMO (F), CONSENSUS (G), RSAT (H), HOMER (I), and STORM (J) with the square-root transformed count of “control” reads in the 400 bp region surrounding each CTCF motif instance.

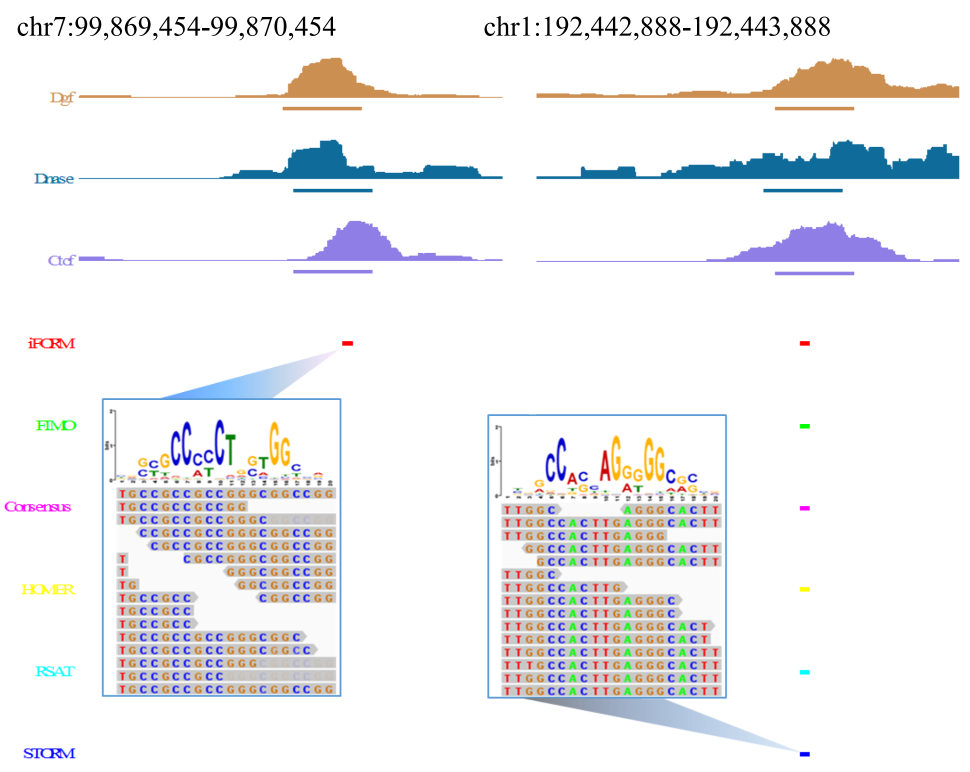

chr7:99,869,454-99,870,454
chr1:192,442,888-192,443,888
Dgf
Dnase
Ctcf
iFORM
FIMO
Consensus
HOMER
RSAT
STORM

Figure A

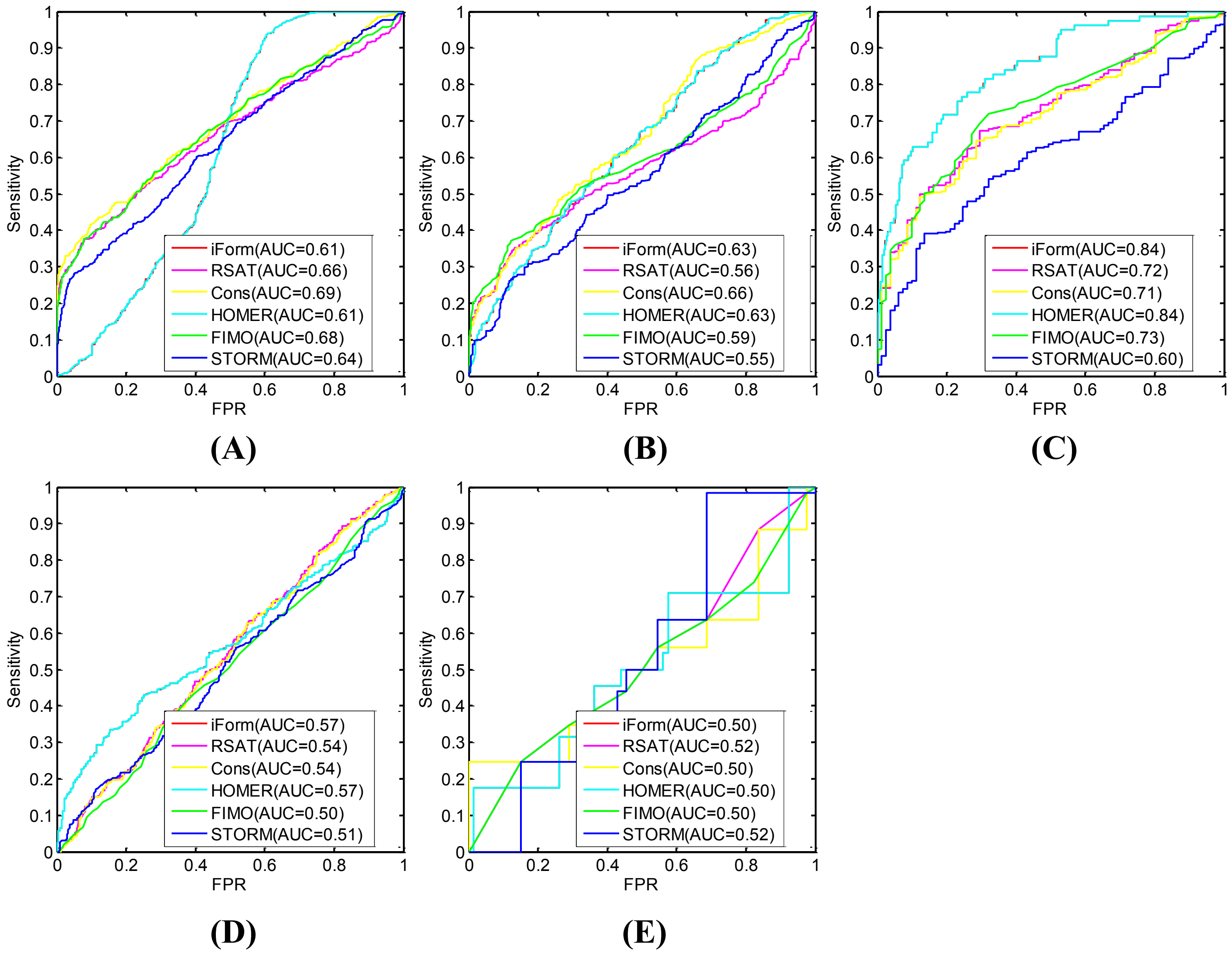
Sensitivity
FPR
iForm(AUC=0.61)
RSAT(AUC=0.66)
Cons(AUC=0.69)
HOMER(AUC=0.61)
FIMO(AUC=0.68)
STORM(AUC=0.64)
(A)
iForm(AUC=0.63)
RSAT(AUC=0.56)
Cons(AUC=0.66)
HOMER(AUC=0.63)
FIMO(AUC=0.59)
STORM(AUC=0.55)
(B)
iForm(AUC=0.84)
RSAT(AUC=0.72)
Cons(AUC=0.71)
HOMER(AUC=0.84)
FIMO(AUC=0.73)
STORM(AUC=0.60)
(C)
iForm(AUC=0.57)
RSAT(AUC=0.54)
Cons(AUC=0.54)
HOMER(AUC=0.57)
FIMO(AUC=0.50)
STORM(AUC=0.51)
(D)
iForm(AUC=0.50)
RSAT(AUC=0.52)
Cons(AUC=0.50)
HOMER(AUC=0.50)
FIMO(AUC=0.50)
STORM(AUC=0.52)
(E)

Figure B

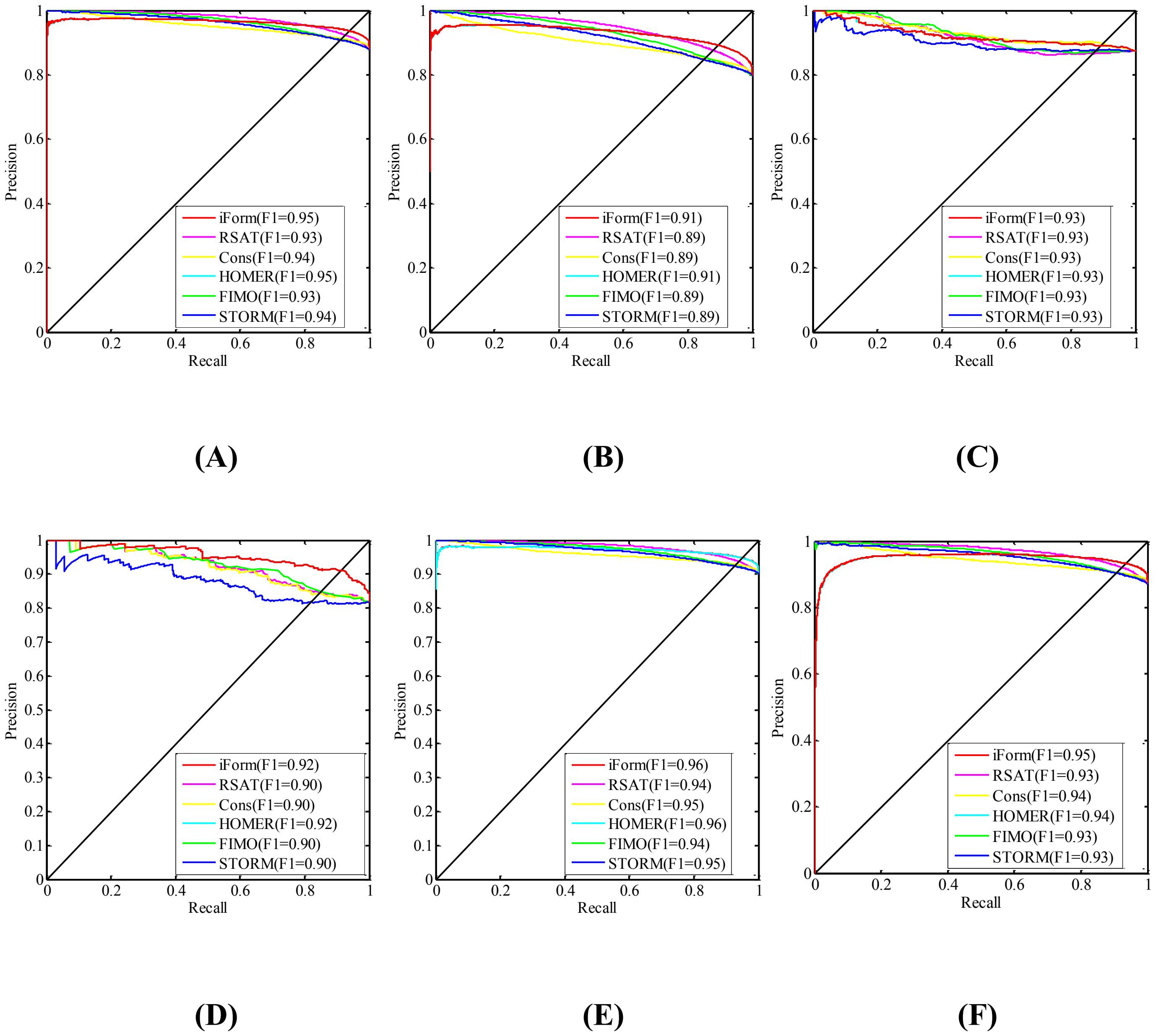

Precision
Recall
iForm(F1=0.95)
RSAT(F1=0.93)
Cons(F1=0.94)
HOMER(F1=0.95)
FIMO(F1=0.93)
STORM(F1=0.94)
(A)
iForm(F1=0.91)
RSAT(F1=0.89)
Cons(F1=0.89)
HOMER(F1=0.91)
FIMO(F1=0.89)
STORM(F1=0.89)
(B)
iForm(F1=0.93)
RSAT(F1=0.93)
Cons(F1=0.93)
HOMER(F1=0.93)
FIMO(F1=0.93)
STORM(F1=0.93)
(C)
iForm(F1=0.92)
RSAT(F1=0.90)
Cons(F1=0.90)
HOMER(F1=0.92)
FIMO(F1=0.90)
STORM(F1=0.90)
(D)
iForm(F1=0.96)
RSAT(F1=0.94)
Cons(F1=0.95)
HOMER(F1=0.96)
FIMO(F1=0.94)
STORM(F1=0.95)
(E)
iForm(F1=0.95)
RSAT(F1=0.93)
Cons(F1=0.94)
HOMER(F1=0.94)
FIMO(F1=0.93)
STORM(F1=0.93)
(F)

Figure C

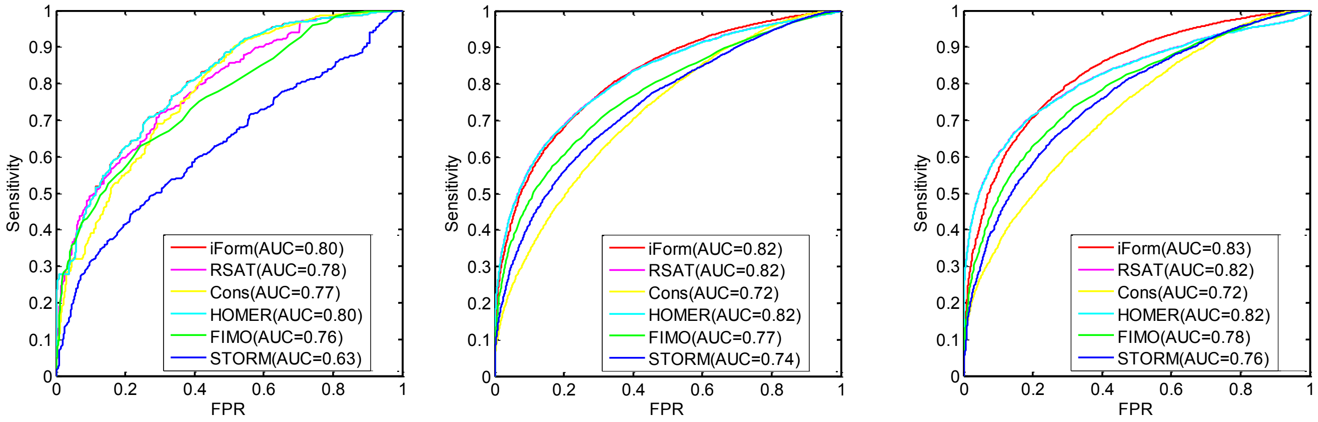
Sensitivity
FPR
iForm(AUC=0.80)
RSAT(AUC=0.78)
Cons(AUC=0.77)
HOMER(AUC=0.80)
FIMO(AUC=0.76)
STORM(AUC=0.63)
iForm(AUC=0.82)
RSAT(AUC=0.82)
Cons(AUC=0.72)
HOMER(AUC=0.82)
FIMO(AUC=0.77)
STORM(AUC=0.74)
iForm(AUC=0.83)
RSAT(AUC=0.82)
Cons(AUC=0.72)
HOMER(AUC=0.82)
FIMO(AUC=0.78)
STORM(AUC=0.76)

(A) **(B)** **(C)**

Figure D

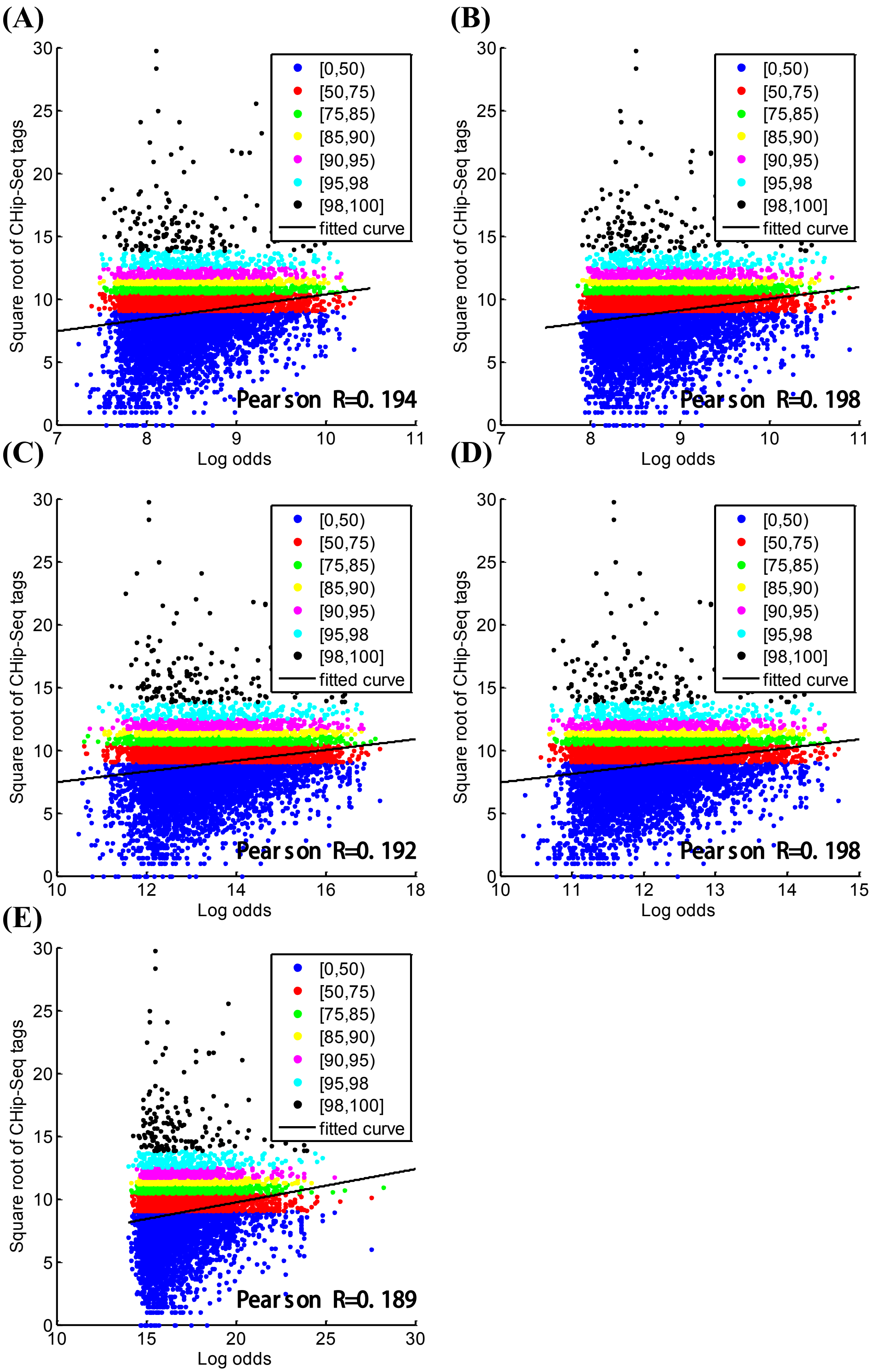
(A)
(B)
(C)
(D)
(E)
Square root of CHip-Seq tags
Log odds
[0,50)
[50,75)
[75,85)
[85,90)
[90,95)
[95,98
[98,100]
fitted curve
Pearson R=0. 194
Pearson R=0. 198
Pearson R=0. 192
Pearson R=0. 198
Pearson R=0. 189

Figure E

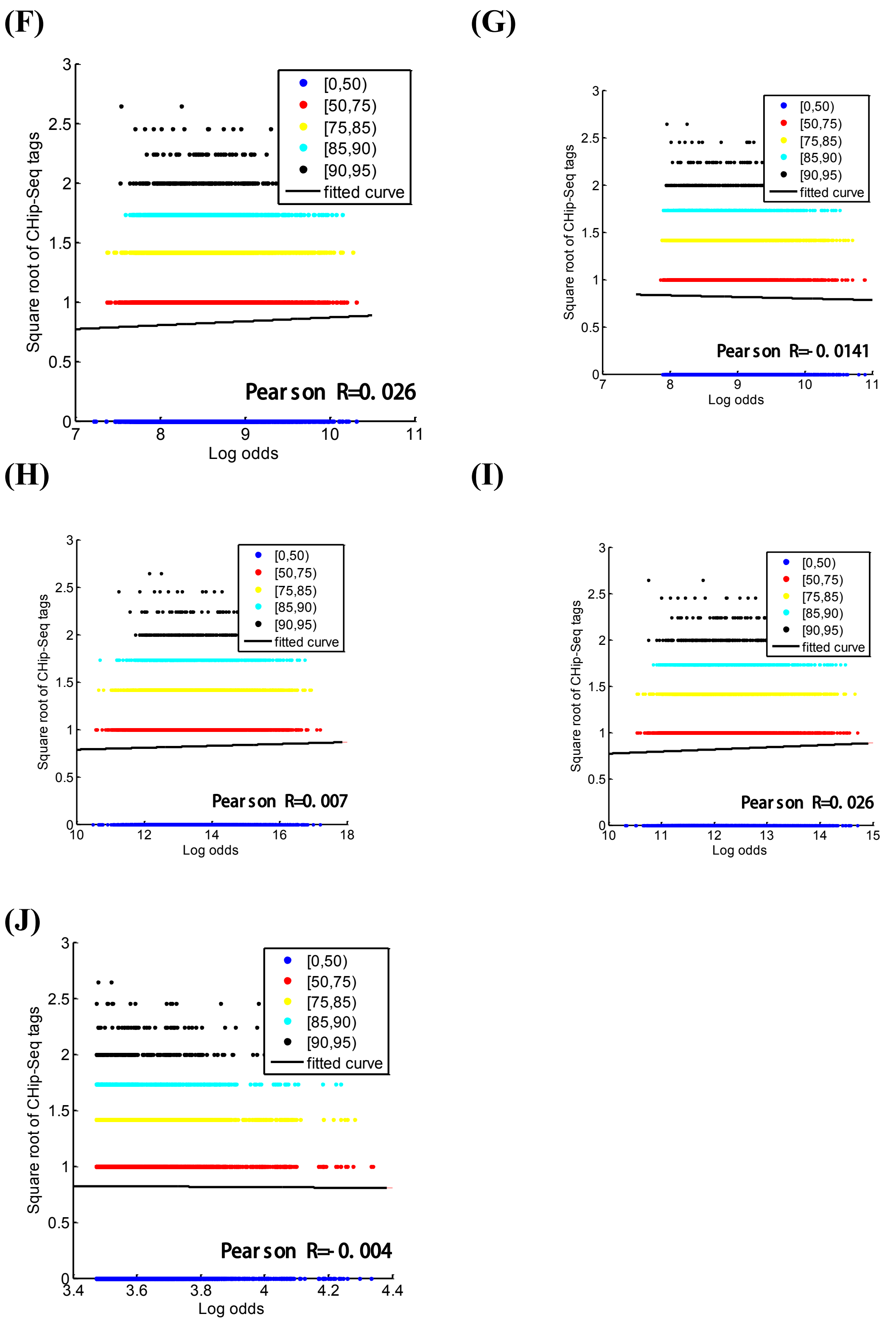
(F)
Square root of CHip-Seq tags
Log odds
[0,50)
[50,75)
[75,85)
[85,90)
[90,95)
fitted curve
Pearson R=0.026
(G)
Square root of CHip-Seq tags
Log odds
[0,50)
[50,75)
[75,85)
[85,90)
[90,95)
fitted curve
Pearson R=-0.0141
(H)
Square root of CHip-Seq tags
Log odds
[0,50)
[50,75)
[75,85)
[85,90)
[90,95)
fitted curve
Pearson R=0.007
(I)
Square root of CHip-Seq tags
Log odds
[0,50)
[50,75)
[75,85)
[85,90)
[90,95)
fitted curve
Pearson R=0.026
(J)
Square root of CHip-Seq tags
Log odds
[0,50)
[50,75)
[75,85)
[85,90)
[90,95)
fitted curve
Pearson R=-0.004

Figure E
