# Supplementary material for: iFORM: Incorporating Find Occurrence of Regulatory Motifs
Source: PLoS One. 2016 Dec 19;11(12):e0168607. doi: 10.1371/journal.pone.0168607 (PMC5167396; doi:10.1371/journal.pone.0168607)
Supplement: S1 Table — (DOCX) [file pone.0168607.s002.docx]

## S1 Table. Summaries of six motif scanners.

| **Method** | **Score** | ***P*-vale** | ***Q*-value** | **programming language** | **Computing time** |
| --- | --- | --- | --- | --- | --- |
| FIMO | $f\left( x \right)={log}_{2}\frac{{letter}_{freq}}{{background}_{freq}}$ | √ | √ | C | faster |
| CONSENSUS | $f\left( x \right)={log}_{e}\frac{{letter}_{freq}}{{col}_{sum}*0.25}$ | √ | × | C | fast |
| HOMER | $f\left( x \right)={log}_{e}\frac{{letter}_{freq}}{0.25}$ | √ | × | C and Perl | normal |
| RSAT | $f\left( x \right)={log}_{e}\left( matrix,{letter}_{freq} \right)-Markov\_logP(bg,{letter}_{freq})$ | √ | × | C, Perl, and Python | normal |
| STORM | $f\left( x \right)={log}_{2}\frac{{letter}_{freq}+base\_comp}{{base}_{comp}*(count+1)}$ | × | × | C | fast |
| iFORM | $\chi^{2}=-2\sum_{i=1}^{5} {log}_{e}\left( p_{i} \right) \sim\chi^{2}(10)$ | √ | √ | C | fast |

Typical features of the five existing motif scanners and iFORM are listed.
